# Motor impairment and compensation in a hemiparkinsonian rat model: correlation between dopamine depletion severity, cerebral metabolism and gait patterns

**DOI:** 10.1186/s13550-017-0317-9

**Published:** 2017-08-23

**Authors:** Elena Kordys, Nadine Apetz, Katharina Schneider, Eilidh Duncan, Beatriz Büschbell, Cathrin Rohleder, Michael Sué, Alexander Drzezga, Bernd Neumaier, Lars Timmermann, Heike Endepols

**Affiliations:** 1 Institute of Radiochemistry and Experimental Molecular Imaging, University Hospital of Cologne, Kerpener Str. 62, 50937 Köln, Germany; 2 Department of Nuclear Medicine, University Hospital of Cologne, Kerpener Str. 62, 50937 Köln, Germany; 3 Max-Planck Institute of Metabolism Research, Gleueler Str. 50, 50931 Köln, Germany; 4 Department of Neurology, University Hospital of Cologne, Kerpener Str. 62, 50937 Köln, Germany; 5 Department of Psychiatry and Psychotherapy, Central Institute of Mental Health, Medical Faculty Mannheim, Heidelberg University, J5, 68159 Mannheim, Germany; 6 Forschungszentrum Jülich GmbH, Institute for Neuroscience and Medicine, INM-5: Nuclear Chemistry, 52425 Jülich, Germany

**Keywords:** Hemiparkinsonian rat model, Positron emission tomography, Dopamine, Brain metabolism, Gait analysis

## Abstract

**Background:**

In Parkinson’s disease (PD), cerebral dopamine depletion is associated with PD subtype-specific metabolic patterns of hypo- and hypermetabolism. It has been hypothesised that hypometabolism reflects impairment, while hypermetabolism may indicate compensatory activity. In order to associate metabolic patterns with pathophysiological and compensatory mechanisms, we combined resting state [^18^F]FDG-PET (to demonstrate brain metabolism in awake animals), [^18^F]FDOPA-PET (dopamine depletion severity) and gait analysis in a unilateral 6-hydroxydopamine rat model.

**Results:**

We found unilateral nigro-striatal dopaminergic loss to decrease swing speed of the contralesional forelimb and stride length of all paws in association with depletion severity. Depletion severity was found to correlate with compensatory changes such as increased stance time of the other three paws and diagonal weight shift to the ipsilesional hind paw. [^18^F]FDG-PET revealed ipsilesional hypo- and contralesional hypermetabolism; metabolic deactivation of the ipsilesional network needed for sensorimotor integration (hippocampus/retrosplenial cortex/lateral posterior thalamus) was solely associated with bradykinesia, but hypometabolism of the ipsilesional rostral forelimb area was related to both pathological and compensatory gait changes. Mixed effects were also found for hypermetabolism of the contralesional midbrain locomotor region, while contralesional striatal hyperactivation was linked to motor impairments rather than compensation.

**Conclusions:**

Our results indicate that ipsilesional hypo- and contralesional hypermetabolism contribute to both motor impairment and compensation. This is the first time when energy metabolism, dopamine depletion and gait analysis were combined in a hemiparkinsonian model. By experimentally increasing or decreasing compensational brain activity, its potential and limits can be further investigated.

## Background

Parkinson’s disease (PD) is characterised by progressive degeneration of the nigrostriatal dopaminergic system which accounts for motor impairments such as bradykinesia and rigidity [1]. Diagnostic PET and SPECT imaging are routinely used to visualise dopaminergic system integrity, using tracers which target either dopamine synthesis and storage capacity (l-3,4-dihydroxy-6-[^18^F]fluorophenylalanine = [^18^F]FDOPA) or dopamine transporter density (e.g. [^123^I]FP-CIT; [2]). Distinct patterns of glucose hyper- and hypometabolism (measured with 2-deoxy-2-[^18^F]fluoroglucose = [^18^F]FDG) are associated with different PD subtypes and correlate with nigrostriatal dopaminergic decline [3, 4]. However, associating focal metabolic changes with specific pathophysiological or compensatory mechanisms is almost impossible as these processes occur simultaneously in both hemispheres. The term ‘compensation’ is used to describe new neuronal activation patterns which result from the adaptation of remaining motor elements and integration of alternative strategies (e.g. recruitment of additional brain regions [5] and adaptation of walking patterns). In contrast, ‘pathophysiological changes’ refers to the metabolic effects of dopamine depletion which are mitigated by recovery, i.e. reacquisition of original functional patterns [5]. In unilateral animal models of PD (‘hemiparkinsonian’ rats), the intact hemisphere may be the primary source of compensatory changes and so helps to disentangle pathology from compensation.

The commonest hemiparkinsonian rat model comprises injection of the neurotoxin 6-hydroxydopamine (6-OHDA) into the nigrostriatal pathway of one hemisphere, producing unilateral degeneration of dopaminergic neurons [6]. Lesion severity can be non-invasively assessed by [^18^F]FDOPA-PET as results correlate well with tyrosine hydroxylase immunohistochemistry [7]. The induced motor phenotype is defined by numerous gait changes such as speed, weight support, stability and paw interaction. The severity of these changes appears to depend on the degree of dopamine depletion [8, 9], as corroborated by [^18^F]FDG-PET which found decreased glucose metabolism in ipsilesional motor brain areas to correlate with apomorphine-induced turning behaviour [10]. Furthermore, ipsilateral hippocampal hypometabolism and bilateral cerebellar hypermetabolism were accompanied by decreasing DAT density as measured by [^18^F]FECT [11].

These results suggest interrelation of striatal dopamine depletion, focal changes of glucose metabolism and motor impairments; however, a study combining all three parameters has not yet been performed. We combined [^18^F]FDOPA-PET, awake resting state [^18^F]FDG-PET and gait analysis in hemiparkinsonian and sham-operated rats to establish whether dopamine depletion severity is correlated with resting state glucose metabolism and walking patterns. This study also provides a baseline for further therapeutic approaches such as dopamine substitution and deep brain stimulation.

## Methods

### Animals

Thirteen adult male Long Evans rats (Janvier, France; 328–365 g, 3 months old) were housed in pairs in type 4 cages with elevated roofs under controlled ambient conditions (22 ± 1 °C and 55 ± 5% relative humidity) on an inversed 12-h light/dark schedule (lights on 8:30 pm–8:30 am). They had free access to water and received 18 g food per animal per day. All experiments took place in the dark (the active phase of rats’ day-night cycle). Experiments were carried out according to German animal protection laws and were approved by the local animal care committee (State Office for Nature, Environment and Consumer Protection of North-Rhine Westphalia, Dept. Animal Welfare).

### Surgery

Animals were anesthetised (initial dosage 5% isoflurane in O_2_/N_2_O (3:7), reduced to 1.5–2.5% isoflurane for maintenance) and received 0.1 ml carprofen (Rimadyl®, Pfizer®, Berlin, Germany) subcutaneously as pain relief. Each animal was fixed on a warming pad in a stereotactic system with a motorised stereotactic drill and injection robot (Robot Stereotaxic, Neurostar®, Tübingen, Germany). Twenty-one micrograms of 6-OHDA in 3 μl NaCl was injected unilaterally into the medial forebrain bundle (*n* = 7; coordinates −4.4 mm posterior, 1.2 mm lateral, 7.9 mm ventral to Bregma). In sham animals (*n* = 6), 3 μl NaCl was injected. Injection side (left/right) was randomised.

A guide cannula (outer diameter 0.71 mm, length 8 mm, PlasticsOne®, Roanoke, VA, USA) was implanted into the injected hemisphere (−3.6 mm posterior, 2.8 mm lateral), targeting the subthalamic nucleus for deep brain stimulation in both 6-OHDA-lesioned and sham animals. During the experiments, the catheter was closed by a nylon filament attached to a cap; no electrode was inserted.

### PET measurements

[^18^F]FDOPA-PET measurements were conducted with tracer uptake under isoflurane anaesthesia between days 26 and 29 after 6-OHDA/sham injection. First, 15 mg/kg benserazide (Sigma-Aldrich; Steinheim, Germany) was injected intraperitoneally to block peripheral decarboxylation of [^18^F]FDOPA. One hour later, 64.4 ± 6.1 MBq of [^18^F]FDOPA in 500 μl NaCl was injected into a lateral tail vein at the start of the 60 min emission scan, followed by a 10-min transmission scan.

After Fourier rebinning, images were reconstructed into two 30-min frames using the iterative OSEM3D/MAP procedure [12] resulting in voxel sizes of 0.38 × 0.38 × 0.82 mm. Only the second frame (30–60 min after [^18^F]FDOPA injection) was used for further analysis. Images were co-registered manually to the Swanson rat brain atlas [13], smoothed with a Gauss kernel of 1.5 mm FWHM and intensity normalised to the cerebellum (SUVR_cer_ = individual voxel value divided by the mean value of cerebellum). Images with right hemispheric injections were flipped, so the intervention was always displayed on the left. Volumes of interest (VOIs) were drawn of the medial and lateral striatum, medial and lateral shell as well as the core of the nucleus accumbens. Ipsi- versus contralesional mean SUVRs were compared using a paired *t* test. In addition, dopamine depletion severity was calculated using the ipsi-/contralesional SUVR ratio of a combined VOI of striatum and nucleus accumbens: dopamine depletion severity = 1 − (SUVR_cer_ipsi/SUVR_cer_contra).

While [^18^F]FDOPA-PET can be used to image dopaminergic system integrity in the anaesthetised animal, [^18^F]FDG uptake must occur in the awake state [14] as anaesthesia strongly decreases cerebral glucose metabolism [15]. Resting state [^18^F]FDG-PET measurements were performed between days 13 and 24 after 6-OHDA injection. 73.0 ± 3.7 MBq of [^18^F]FDG in 500 μl NaCl was injected intraperitoneally into the awake animal, which was then transferred to a dark chamber of 30 × 24 × 21 cm equipped with a video camera (Logitech webcam) and infrared LEDs. The rat was watched during [^18^F]FDG uptake to make sure it did not sleep. The resulting conditions (awake and free to move, without sensory stimulation or an assigned task) will subsequently be called ‘resting state’. After 45 min in the chamber, the animal was anesthetised (initial dosage 5% isoflurane in O_2_/air (3:7), reduced to 1.5–2.5% isoflurane for maintenance) and fixed on an animal holder (Medres®, Cologne, Germany) with a respiratory mask. Body temperature was maintained at 37 °C using a feedback-controlled warming system (Medres®). A 30-min emission scan was started 60 min after [^18^F]FDG injection using a Focus 220 micro-PET scanner (CTI-Siemens). This was followed by a 10-min transmission scan using a ^57^Co point source for attenuation correction. Blood glucose concentration was measured at the end of each [^18^F]FDG emission scan, using a glucose level meter with test strips (Medtronic Contour Link).

Summed images (over 60 to 90 min after [^18^F]FDG injection) were reconstructed as described above. Images were co-registered manually to the Swanson rat brain atlas [13] and intensity normalised to whole brain activity (standardised uptake value ratio SUVR_wb_ = individual voxel value divided by the mean value of the whole brain). Images with right hemispheric injections were flipped, so the intervention was always displayed on the left. Groups (6-OHDA and sham) were compared voxel-wise using a *t* test followed by a threshold-free cluster enhancement (TFCE) procedure with subsequent permutation testing, yielding a statistical map corrected for multiple testing, thresholded at *p* < 0.05 [16].

[^18^F]FDG-PET images of all animals were correlated voxel-wise with dopamine depletion severity using the Pearson correlation analysis. This was followed by a TFCE procedure with subsequent permutation testing. The resulting statistical map was thresholded at *p* < 0.05 (corrected) for visual comparison with the [^18^F]FDG-PET difference map (6-OHDA versus sham) and at *p* < 0.01 (corrected) for visualisation of the most significant correlations.

### Gait analysis

Catwalk XT 10.5 (Noldus®, Wageningen, Netherlands) was used for gait analysis between days 27 and 30 after 6-OHDA/sham injection and between 4 and 15 days after [^18^F]FDG-PET. The analysed walkway section was 39 cm long. Nine compliant runs were recorded per animal (compliant runs did not exceed 5 s with a maximum speed variation of 60%). The following parameters were measured: average speed, body speed for each paw, swing duration, stance duration, stride length, print area and cadence (number of steps per second). Gait parameters were compared using a two-way mixed design ANOVA with factors: treatment (6-OHDA and sham) and paw (contralesional front (CF), contralesional hind (CH), ipsilesional front (IF) and ipsilesional hind (IH)). In addition, Pearson correlations between [^18^F]FDG uptake and gait changes were determined for selected brain areas which showed glucose metabolism to be altered in line with dopamine depletion severity. VOIs were generated from the significant clusters (*p* < 0.01, corrected) found in the ipsilesional medial striatum, contralesional caudal striatum (cStr), ipsilesional rostral forelimb area (RFA), contralesional lobulus V of the cerebellum (CerV) and contralesional midbrain locomotor region (MLR).

## Results

### Imaging

**Fig. 1:**
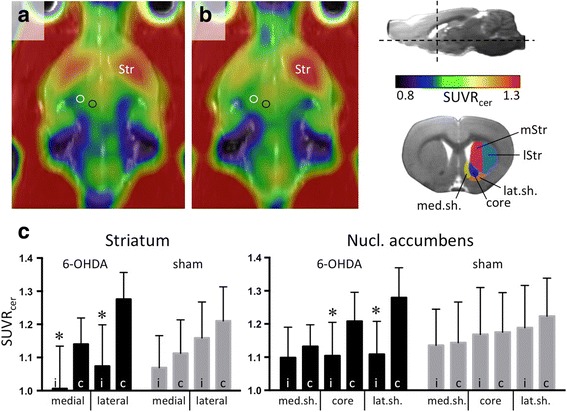
[^18^F]FDOPA imaging in 6-OHDA- and sham-injected rats. **a** Horizontal view of average [^18^F]FDOPA images over *n* = 6 sham (**a**) and *n* = 7 6-OHDA-injected rats (**b**). Images with injections on the right side were flipped so that all injections are displayed on the left. The white circle shows the location of the implanted guide cannula for the stimulation electrode (electrode was not inserted in this study). The black circle shows the 6-OHDA or saline injection site. Horizontal and transverse section planes are indicated in the upper right corner. VOIs are shown below. **c** Mean [^18^F]FDOPA SUVR_cer_ values (with standard deviation) from the VOIs displayed in the insert. Asterisks: significant differences (paired *t* test, *p* < 0.05) between ipsi- and contralesional VOIs. Abbreviations: c contralesional, core core of nucleus accumbens, i ipsilesional, lat.sh. lateral shell of nucleus accumbens, lStr lateral striatum, med.sh. medial shell of nucleus accumbens, mStr medial striatum, Str striatum, SUVR_cer_ standardised uptake value ratio with cerebellum as reference

Four weeks after 6-OHDA injection [^18^F]FDOPA uptake was significantly reduced in the ipsilesional medial and lateral striatum as well as in the nucleus accumbens core and lateral shell, as compared to respective contralesional areas (Fig. 1). In sham animals, differences between ipsi- and contralateral striatal regions were not significant.

**Fig. 2:**
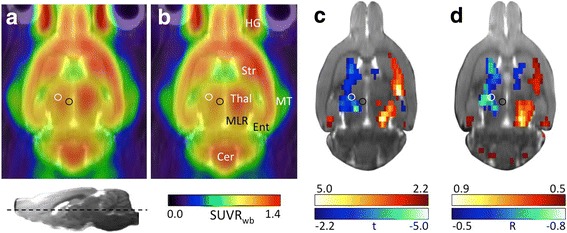
[^18^F]FDG imaging in 6-OHDA- and sham-injected rats. Horizontal views of average [^18^F]FDG images of sham- (**a**) and 6-OHDA-injected rats (**b**). Images with injections on the right side were flipped so that all injections are displayed on the left. The white circle shows the location of the implanted guide cannula for the stimulation electrode (electrode was not inserted in this study). The black circle shows the 6-OHDA or saline injection site. The horizontal section plane is indicated in the l*ower left* corner. **c** Subtractive t-map (*p* < 0.05, TFCE-corrected) displaying significant differences between both groups. *Red* colour shows voxels where [^18^F]FDG accumulation was higher in 6-OHDA rats compared to shams. *Blue* voxels indicate a lower [^18^F]FDG accumulation in 6-OHDA rats compared to shams. **d** R-map (*p* < 0.05, TFCE-corrected) showing significant correlation between glucose metabolism and dopamine depletion severity. Red: voxels with positive correlation, i.e. [^18^F]FDG accumulation increases with increasing depletion severity. *Blue*: voxels with negative correlation, i.e. [^18^F]FDG accumulation decreases with increasing depletion severity. Abbreviations: Cer cerebellum, Ent entorhinal cortex, HG Harderinan gland, MLR midbrain locomotor region, MT medial temporal muscle, Str striatum, SUVR_wb_ standardised uptake value ratio with the whole brain as reference, Thal thalamus

Blood glucose concentration was 8.06 ± 0.90 mmol/L in lesioned and 7.87 ± 1.11 mmol/L in sham animals. [^18^F]FDG uptake, measured 2 to 3 weeks after 6-OHDA injection, was significantly reduced in ipsilesional subcortical areas, including the medial striatum and thalamus, when compared to sham animals. In the contralesional hemisphere, [^18^F]FDG uptake was significantly increased in the lateral and caudal striatum and the midbrain reticular formation (Fig. 2), showing 6-OHDA injection to have caused a subcortical imbalance between hemispheres with ipsilesional hypo- and contralesional hypermetabolism.

**Fig. 3:**
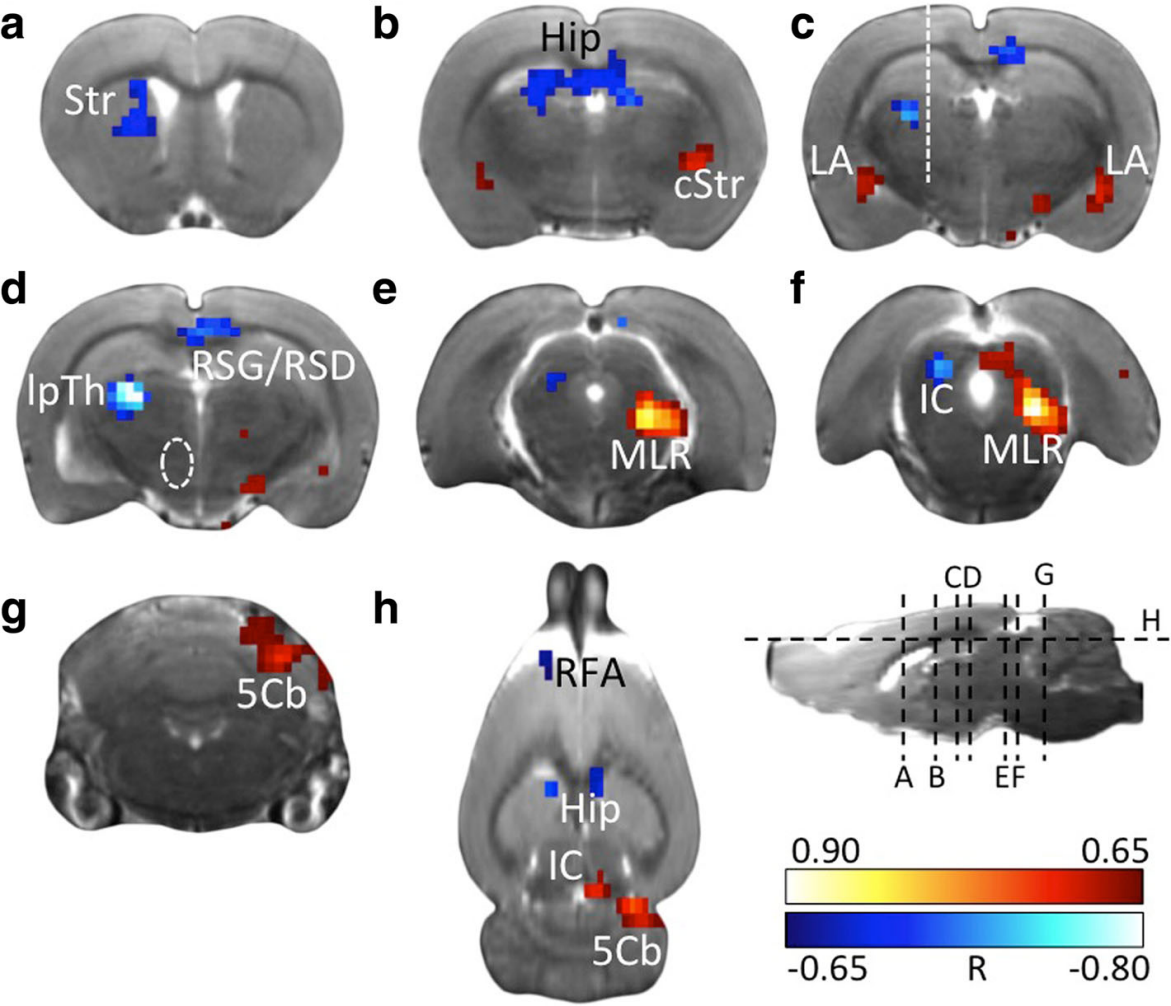
Correlation between lesion severity and brain metabolism. Transverse sections (**a**–**g**) and horizontal section (**h**) showing significant correlation (*p* < 0.01, TFCE-corrected) between [^18^F]FDG accumulation and dopamine depletion severity. Section levels are indicated by dashed lines in the sagittal section. White dashed line in **c**: Location of guide cannula. White dashed line in **d**: 6-OHDA injection site. Red: voxels with positive correlation, i.e. metabolism increases with increasing lesion severity. Blue: voxels with negative correlation, i.e. metabolism decreases with increasing lesion severity. The colour scale shows the correlation coefficient R. Abbreviations: 5Cb cerebellar lobule V, cStr caudal striatum, Hip hippocampus, IC inferior colliculus, LA lateral amygdala, lpTh lateral posterior thalamus, MLR midbrain locomotor region, RSG/RSD retrosplenial granular/dysgranular cortex, Str striatum

[^18^F]FDG uptake was correlated with dopamine depletion severity and followed the same pattern as the metabolic differences between hemiparkinsonian and sham animals (Figs. 2 and 3a–h). Negative correlations, indicating decreasing metabolism with increasing depletion severity, were mainly found in the ipsilesional hemisphere: rostral forelimb area (RFA), mediodorsal striatum, lateral posterior thalamus (lpTh), dorsolateral geniculate nucleus and inferior colliculus. In the retrosplenial cortex (RSC) and anterior dorsal hippocampus, negative correlations were bilateral.

Positive correlations, indicating increasing metabolism with increasing dopamine depletion severity, occurred predominantly in the contralesional hemisphere and affected the caudolateral striatum, midbrain locomotor region (MLR: deep mesencephalic nucleus, cuneiform nucleus), inferior colliculus and cerebellar lobule V. In the lateral amygdala, positive correlations were bilateral.

### Gait analysis

**Fig. 4:**
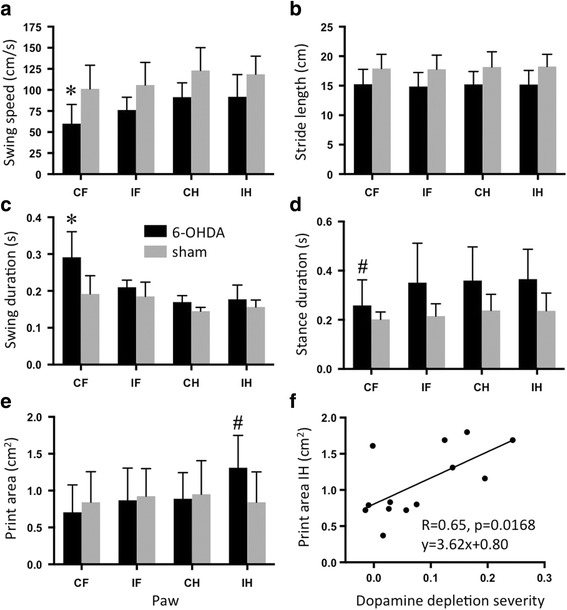
Gait parameters of 6-OHDA and sham injected rats. Swing speed (**a**), stance duration (**b**), swing duration (**c**) and print area (**d**) were significantly altered after 6-OHDA injection. **e** Stride length was generally decreased (significant main effect for factor treatment but no significant post hoc differences). **f** Correlation between IH print area and dopamine depletion. Asterisk indicates significant difference between 6-OHDA and sham for the indicated paw. Number sign indicates significant difference between the indicated paw and all other paws within the 6-OHDA group. Depletion severity = 1 − (SUVR_cer_ipsi/SUVR_cer_contra). Abbreviations: CF contralesional forelimb, CH contralesional hindlimb, IF ipsilesional forelimb, IH ipsilesional hindlimb

**Fig. 5:**
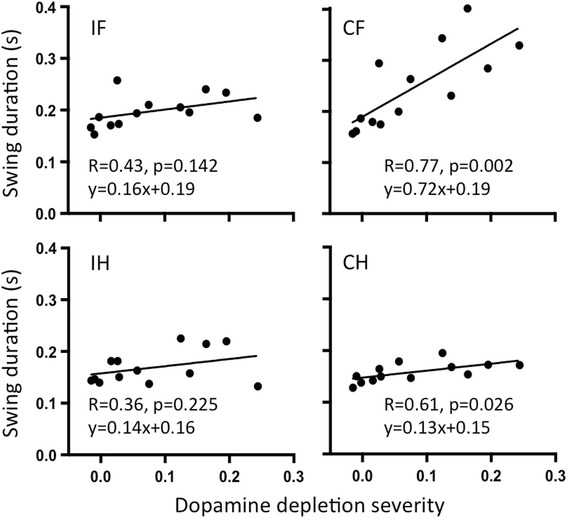
Correlation between swing duration and dopamine depletion severity. Shown are correlation coefficients *R* and linear equations of regression lines. *P* values are the same for correlation and regression. Depletion severity = 1 − (SUVR_cer_ipsi/SUVR_cer_contra). Abbreviations: CF contralesional forelimb, CH contralesional hindlimb, IF ipsilesional forelimb, IH ipsilesional hindlimb

Four weeks after injection, the average walking speed of hemiparkinsonian animals (29 ± 14 cm/s) was significantly reduced compared to sham rats (46 ± 13 cm/s; *p* = 0.0498). This was caused by a reduction in CF swing speed (*F*(1,11) = 7.2, *p* = 0.0213 for factor treatment; significant differences between 6-OHDA and sham for CF only; Fig. 4a). A general reduction in stride length contributed to the decrease in walking speed (*F*(1,11) = 5.1, *p* = 0.0451 for factor treatment, no significant differences for post hoc testing; Fig. 4b). Both CF swing speed and CF swing duration (Fig. 5) correlated strongly with dopamine depletion severity (*R* = −0.81, *p* = 0.0007 and *R* = 0.77, *p* = 0.0023, respectively). Swing duration of the other three paws remained at sham level (Fig. 4c), while their stance duration was significantly higher than CF (*F*(3,33) = 12.5, *p* < 0.0001 for factor paw; *F*(3,33) = 3.8, *p* = 0.0194 for interaction between paw and treatment; significant difference between CF and all other paws within 6-OHDA; Fig. 4d). Swing speed, stance duration, stride length and body speed of all paws correlated with dopamine depletion severity (Table 1).

**Table 1:** Correlation between gait parameters and dopamine depletion severity, measured with [^18^F]FDOPA

		Contralesional front paw	Contralesional hind paw	Ipsilesional front paw	Ipsilesional hind paw
Swing duration	s	*R* = 0.77 *p* = 0.0023**	*R* = 0.61 *p* = 0.0256*	*R* = 0.43 *p* = 0.1416	*R* = 0.36 *p* = 0.2252
Swing speed	cm/s	*R* = −0.81 *p* = 0.0007**	*R* = −0.72 *p* = 0.0057**	*R* = −0.75 *p* = 0.0034**	*R* = −0.66 *p* = 0.0132*
Stance duration	s	*R* = 0.78 *p* = 0.0016**	*R* = 0.81 *p* = 0.0008**	*R* = 0.85 *p* = 0.0002**	*R* = 0.87 *p* = 0.0001**
Stride length	cm	*R* = −0.79 *p* = 0.0004**	*R* = −0.83 *p* = 0.0004**	*R* = −0.80 *p* = 0.0010**	*R* = −0.82 *p* = 0.0006**
Body speed	cm/s	*R* = −0.77 *p* = 0.0020**	*R* = −0.79 *p* = 0.0014**	*R* = −0.80 *p* = 0.0009**	*R* = −0.80 *p* = 0.0011**
Print area	cm^2^	*R* = 0.05 *p* = 0.8719	*R* = 0.13 *p* = 0.6645	*R* = 0.19 *p* = 0.5247	*R* = 0.65 *p* = 0.0168*
		Global			Global
Average speed	cm/s	*R* = −0.81 *p* = 0.0007**		Number of steps	*R* = 0.88 *p* = 0.0001**
Cadence	steps/s	*R* = −0.79 *p* = 0.0012**			

Positive correlations: strong dopamine depletion is associated with high behavioural value. Negative correlations: strong dopamine depletion is associated with low behavioural value. *p < 0.05; **p < 0.01

IH showed an increase in print area (*F*(3,33) = 4.6, *p* < 0.0088 for factor paw; *F*(3,33) = 5.7, *p* = 0.0028 for interaction between paw and treatment, significant difference between IH and all other paws within 6-OHDA; Fig. 4e), which also correlated significantly with dopaminergic lesion severity (*R* = 0.65, *p* = 0.0168; Fig. 4f).

Correlations between [^18^F]FDG uptake and gait changes were determined for brain areas which showed glucose metabolism to be altered in line with dopamine depletion severity. Given that severity of impairment and strength of compensation are interrelated, weak to medium correlations were expected between metabolic changes of both processes at the same time. For this reason, we will focus on either strong (i.e. *R* > 0.68) or singular (i.e. to the affected paw only) correlations. For an overview of correlation results see Table 2.

**Table 2:** Correlation between gait parameters and metabolic activity in selected brain areas, measured with [^18^F]FDG (*n* = 13)

		Contralesional front paw	Contralesional hind paw	Ipsilesional front paw	Ipsilesional hind paw
RFA, ipsilesional					
Swing speed	cm/s	*R* = 0.67 *p* = 0.0119*	*R* = 0.62 *p* = 0.0239*	*R* = 0.62 *p* = 0.0224*	*R* = 0.60 *p* = 0.0300*
Swing duration	s	*R* = −0.59 *p* = 0.0355*	*R* = −0.47 *p* = 0.1065	*R* = −0.41 *p* = 0.1660	*R* = −0.38 *p* = 0.1966
Stance duration	s	*R* = −0.54 *p* = 0.0577	*R* = −0.63 *p* = 0.0209*	*R* = −0.64 *p* = 0.0194*	*R* = −0.64 *p* = 0.0187*
Stride length	cm	*R* = 0.59 *p* = 0.0343*	*R* = 0.64 *p* = 0.0190*	*R* = 0.55 *p* = 0.0496*	*R* = 0.61 *p* = 0.0272*
Print area	cm^2^	*R* = −0.16 *p* = 0.6118	*R* = −0.16 *p* = 0.6051	*R* = −0.31 *p* = 0.3030	*R* = −0.69 *p* = 0.0087**
Body speed	cm/s	*R* = 0.64 *p* = 0.0176*	*R* = 0.68 *p* = 0.0110*	*R* = 0.66 *p* = 0.0141*	*R* = 0.67 *p* = 0.0120*
lpTh, ipsilesional					
Swing speed	cm/s	*R* = 0.69 *p* = 0.0084**	*R* = 0.71 *p* = 0.0070**	*R* = 0.67 *p* = 0.0115*	*R* = 0.66 *p* = 0.0139*
Stance duration	s	*R* = −0.63 *p* = 0.0208*	*R* = −0.61 *p* = 0.0270*	*R* = −0.65 *p* = 0.0155*	*R* = −0.65 *p* = 0.0155*
Stride length	cm	*R* = 0.70 *p* = 0.0082**	*R* = 0.74 *p* = 0.0041**	*R* = 0.69 *p* = 0.0087**	*R* = 0.75 *p* = 0.0030**
Body speed	cm/s	*R* = 0.69 *p* = 0.0094**	*R* = 0.71 *p* = 0.0071**	*R* = 0.71 *p* = 0.0062**	*R* = 0.71 *p* = 0.0066**
cStr, contralesional					
Swing speed	cm/s	*R* = −0.70 *p* = 0.0072**	*R* = −0.79 *p* = 0.0013**	*R* = −0.67 *p* = 0.0117*	*R* = −0.71 *p* = 0.0065**
Swing duration	s	*R* = 0.53 *p* = 0.0613	*R* = 0.71 *p* = 0.0067**	*R* = 0.35 *p* = 0.2454	*R* = 0.49 *p* = 0.0888
Stance duration	s	*R* = 0.68 *p* = 0.0100*	*R* = 0.64 *p* = 0.0173*	*R* = 0.73 *p* = 0.0047**	*R* = 0.71 *p* = 0.0071**
Stride length	cm	*R* = −0.74 *p* = 0.0038**	*R* = −0.76 *p* = 0.0023**	*R* = −0.74 *p* = 0.0042**	*R* = −0.82 *p* = 0.0006**
Print area	cm^2^	*R* = 0.05 *p* = 0.8757	*R* = 0.17 *p* = 0.5808	*R* = 0.54 *p* = 0.0559	*R* = 0.66 *p* = 0.0146*
Body speed	cm/s	*R* = −0.72 *p* = 0.0059**	*R* = −0.76 *p* = 0.0028**	*R* = −0.77 *p* = 0.0023**	*R* = −0.75 *p* = 0.0033**
CerV, contralesional					
Stance duration	s	*R* = 0.59 *p* = 0.0320*	*R* = 0.44 *p* = 0.1360	*R* = 0.59 *p* = 0.0354*	*R* = 0.50 *p* = 0.0833
Stride length	cm	*R* = −0.45 *p* = 0.1187	*R* = −0.55 *p* = 0.0529	*R* = −0.44 *p* = 0.1310	*R* = −0.58 *p* = 0.0388*
MLR, contralesional					
Swing speed	cm/s	*R* = −0.63 *p* = 0.0223*	*R* = −0.64 *p* = 0.0196*	*R* = −0.56 *p* = 0.045*	*R* = −0.51 *p* = 0.0724
Swing duration	s	*R* = 0.49 *p* = 0.0884	*R* = 0.63 *p* = 0.0188*	*R* = 0.29 *p* = 0.3419	*R* = 0.24 *p* = 0.4254
Stance duration	s	*R* = 0.66 *p* = 0.0145*	*R* = 0.57 *p* = 0.0436*	*R* = 0.66 *p* = 0.0137*	*R* = 0.66 *p* = 0.0142*
Stride length	cm	*R* = −0.61 *p* = 0.0263*	*R* = −0.70 *p* = 0.0078**	*R* = −0.61 *p* = 0.0261*	*R* = −0.70 *p* = 0.0077**
Print area	cm^2^	*R* = 0.20 *p* = 0.5064	*R* = 0.16 *p* = 0.6111	*R* = 0.35 *p* = 0.2478	*R* = 0.60 *p* = 0.0315*
Body speed	cm/s	*R* = −0.59 *p* = 0.0346*	*R* = −0.62 *p* = 0.0248*	*R* = −0.62 *p* = 0.0242*	*R* = −0.62 *p* = 0.0234*

Positive correlations ipsilateral: low metabolism is associated with a low behavioural value. Positive correlations contralateral: high metabolism is associated with a high behavioural value. Negative correlations ipsilateral: low metabolism is associated with a high behavioural value. Negative correlations contralateral: high metabolism is associated with a low behavioural value. *p < 0.05; **p < 0.01

*Abbreviations*: *CerV* cerebellum, lobulus V, *lpTh* lateral posterior thalamic nucleus, *MLR* midbrain locomotor region, *RFA* rostral forelimb area

Increased CF swing duration was found to correlate with a decrease in ipsilesional RFA metabolism (*R* = −0.59, *p* = 0.0355). Decreased CF and CH swing speed were associated with decreasing ipsilesional lpTh metabolism (*R* > 0.69, *p* < 0.0084) and increasing contralesional metabolism of the caudal striatum (cStr; *R* < −0.70, *p* < 0.0072). Decreased stride length of all paws correlated with hypometabolism of the ipsilesional lpTh (*R* > 0.69, *p* < 0.0087) and hypermetabolism of the contralesional cStr (*R* < −0.74, *p* < 0.0038), while decreased stride length of the hind limbs alone correlated strongly with hypermetabolism of the contralesional MLR (*R* = −0.70, *p* < 0.0078). Decreased body speed, as measured for all paws, correlated strongly with decreased metabolism of lpTh (*R* > 0.69, *p* < 0.0094) and increased metabolism of cStr (*R* < −0.72, *p* < 0.0059). Increased stance duration of all paws correlated with increased contralesional cStr metabolism (*R* > 0.64, *p* < 0.0173); increased IH print area however correlated with decreased ipsilateral RFA metabolism (*R* = −0.69, *p* = 0.0087). Hypometabolism of the contralesional CerV exhibited only weak correlations, and no gait changes were associated with metabolic activity in the ipsilesional medial striatum.

## Discussion

Altered brain metabolism and gait abnormalities were found to correlate with unilateral dopaminergic lesion severity in hemiparkinsonian rats, indicating that striatal dopamine depletion, focal changes of glucose metabolism and motor impairments are indeed interrelated. Importantly, CF, which is controlled by the ipsilesional motor cortico-striatal loop, was most affected by bradykinesia. Dopamine depletion significantly decreased CF swing speed in association with depletion severity, as supported by the hypokinesia and rigidity found in human PD patients [17]. Another symptom typical of human PD is reduced stride length [18] which was uniformly decreased across all paws and correlated with depletion severity in our unilateral rat model. Other lesion-related gait changes, particularly on the ipsilesional side, were most likely compensatory in nature (i.e. they represent an alternative behavioural strategy for gait stabilisation); for example, the three less affected paws maintain sham level swing duration but with increased stance duration to meet the increased CF swing time. CF stance duration was significantly shorter compared to the other paws, indicating that CF was partly relieved from weight loading and propulsion. Consequently, body weight was shifted to IH, which contacts the ground at the same time as CF during the commonest diagonal gait; this weight shift was reflected by an increased IH print area. These results show how intact interlimb-coordination is utilised to compensate for CF impairment.

The metabolic pattern emerging in correlation with dopamine depletion in rats included regions homologous to those defining the human ‘Parkinson’s disease-related pattern’ (PDRP), which also correlates with declining striatal dopamine [3, 19]. While human PDRP is symmetrical due to bilateral dopaminergic loss, in our hemiparkinsonian rat model, hypometabolism was mostly confined to the ipsilesional and hypermetabolism to the contralesional hemisphere, in line with previous findings [11]. In the ipsilesional RFA, metabolic activity decreased with increasing dopamine depletion, similar to the human frontal premotor areas [20]. In the contralesional hemisphere, metabolic activity increased in the caudal lateral striatum (cStr), pallidum, pedunculopontine tegmental nucleus (PPTg) and cerebellum, as found in the respective PDRP areas [3, 20]. The only difference between our results and human PDRP concerned the posterior parietal cortex where human Parkinson’s patients show a decline in metabolic activity but the area remained unchanged in rats.

Hypometabolic patterns are generally thought to represent dysfunctional areas directly or indirectly associated with PD motor symptoms [21]. The ipsilesional RFA and ipsilesional medial striatum are two regions with apparent motor functions which showed metabolic activity to decrease with increasing dopamine depletion severity. The ipsilesional striatal metabolic decline was not correlated with gait changes. Ipsilesional RFA, however, was the only area where hypometabolism correlated with increased CF swing duration; furthermore, it showed the highest correlation with increased IH print area, i.e. with a compensatory weight shift. This suggests RFA to be involved in CF impairment but it may also be responsible for triggering compensatory changes, possibly via intensive contralateral connections [22].

The highest correlation between metabolic decline and dopamine depletion was found in the ipsilesional lpTh. Furthermore, decreased swing speed, stride length and body speed correlated highly with lpTh hypometabolism. The lpTh is homologous to the pulvinar in other mammals and sends projections to the central and medial striatum [23]; it receives input from the RSC which is in turn innervated by the hippocampus [24], both of which also displayed ipsilesional hypometabolism in our hemiparkinsonian rats. LpTh and RSC are involved in visual attention and have been associated with visuospatial problems in PD patients (e.g. neglect, visual hallucinations and blind-to-blindsight syndrome; [25, 26]). In rats, lpTh and RSC are not only part of the head-direction system but also contain cells which fire in relation to walking speed [27, 28]. The dorsal hippocampus is known for place cells and spatial functions needed for sensorimotor integration [29]. However, the affected network seems to be dedicated not just to ‘passive’ spatial coding but is thought to provide the motor system with either a ‘readiness’ signal for the preparation of movement or a signal relaying the intensity of an intended or ongoing movement [30]. Decreased activity in this network indicates impaired fine tuning of sensorimotor integration in the ipsilesional hemisphere, which may contribute to pathological gait changes.

Hypermetabolic patterns in PD patients are often interpreted as compensatory changes [31] and so, assuming this to be true for our rat model, we would expect strong correlations with compensatory gait changes (increased IH print area and duration of CH, IF and IH stance). Hypermetabolism was seen in the contralesional MLR, cStr, CerV and bilateral amygdala, with the strongest correlation between metabolic activity and dopamine depletion severity occurring in the MLR.

The MLR is needed for initiation and cessation of locomotion [32], and its activity is associated with the speed of movement [33]. It is also where information from the motor cortex, basal ganglia, hypothalamic locomotor region, cerebellum and amygdala converges and is transmitted to the spinal central pattern generators via the pontine reticular formation. However, it is not clear if this activation is associated with compensation as the greatest correlation between MLR hypermetabolism and gait was found in hindlimb stride length reduction, followed by increased ipsilesional stance duration. These mixed effects suggest MLR hyperactivation to be the result of increased excitatory synaptic inputs and loss of inhibition (either in the MLR itself or in its afferent areas), possibly affecting different neuron populations [34].

MLR hypermetabolism may have been triggered by the hyperactive cStr, leading to MLR disinhibition via basal ganglia output nuclei [34]. CStr hypermetabolism was strongly associated with several pathological gait changes (decreased swing speed, stride length and body speed) but with only one compensatory effect (increased stance duration). This suggests that the contralesional basal ganglia network is crucial for bilateral pathological gait changes; it can influence the contralesional spinal motor system through ipsilateral connections (via MLR) and the ipsilesional motor system through contralateral connections (via the basal ganglia-thalamus-cortex loop and crossed corticospinal tract).

It has been suggested that the cerebellum integrates real-time sensory information for motor control [35], which is particularly important when one paw is impaired. However, as CerV was only weakly correlated with gait parameters, it is unlikely to play a major role in our observed behavioural changes.

## Conclusions

Nigro-striatal dopamine depletion, cerebral metabolic patterns and gait changes are intimately connected in hemiparkinsonian rats. Unilateral nigro-striatal dopaminergic loss mainly impairs the contralesional forelimb in line with dopamine depletion severity, while the other paws adapt to enable stable walking. Hypoactivity of the ipsilesional network for sensorimotor integration (hippocampus/retrosplenial cortex/lateral posterior thalamus) was solely associated with bradykinesia, but hypometabolism of the ipsilesional rostral forelimb area RFA was related to both pathological and compensatory gait changes. Mixed effects were also found for hypermetabolism of the contralesional midbrain locomotor region MLR, and contralesional striatal hyperactivation was linked to motor impairments rather than to compensation. Our results indicate that both ipsilesional hypo- and contralesional hypermetabolism contribute to motor impairments as well as to compensation. By experimentally increasing or decreasing compensational brain activity, e.g. by deep brain stimulation, its potential and limits can be further investigated. This animal model may provide the basis for therapeutic strategies strengthening compensation on the level of brain networks in human PD patients.

## Abbreviations

[^18^F]FDG: 2-Deoxy-2-[^18^F]fluoroglucose
[^18^F]FDOPA: l-3,4-Dihydroxy-6-[^18^F]fluorophenylalanine
6-OHDA: 6-Hydroxydopamine
ANOVA: Analysis of variance
CerV: Contralesional lobulus V of the cerebellum
CF: Contralesional front paw
CH: Contralesional hind paw
cStr: Caudal striatum
FWHM: Full width half maximum
IF: Ipsilesional front paw
IH: Ipsilesional hind paw
lpTh: Lateral posterior thalamus
MLR: Midbrain locomotor region
PD: Parkinson’s disease
PDRP: Parkinson’s disease-related pattern
PET: Positron emission tomography
PPTg: Pedunculopontine tegmental nucleus
RFA: Rostral forelimb area
RSC: Retrosplenial cortex
SUVR: Standardised uptake value ratio
TFCE: Threshold-free cluster enhancement
VOI: Volume of interest
